# Determinants of difficult laryngoscopy based on upper airway indicators: a prospective observational study

**DOI:** 10.1186/s12871-024-02543-4

**Published:** 2024-04-24

**Authors:** Jing Yuan, Hui Ye, Xiaoxiang Tan, Hui Zhang, Jie Sun

**Affiliations:** Department of Anesthesiology, ZhongDa Hospital, Southeast University, No. 87 Dingjiaqiao, Gulou District, Nanjing, 210009 China

**Keywords:** Difficult laryngoscopy, Upper airway indicators, Ultrasonography, Risk factors, Predictive performanc

## Abstract

**Background:**

The main cause of anesthesia-related deaths is the failure to manage difficult airways. Difficult laryngoscopic exposure is a major cause of unsuccessful management of difficult airways. Inadequate preoperative airway assessment hinders the clinical management of difficult airways cases, emphasizing the critical need for accurate identification of difficult airways. Currently, no definitive and reliable indicators are available to predict a difficult airway. Our study aims to predict laryngoscope exposure risk factors by combining ultrasonically measured upper airway anatomic parameters with physical examination indicators.

**Methods:**

Patients aged 18 to 75 years, classified as American Standards Association (ASA) I-III, and scheduled for elective general anesthesia with endotracheal intubation were included. All patients received the upper airway and ultrasonographic measurements. After anesthesia induction, laryngoscope exposure was analyzed using the Cormack-Lehane grading system, with Grades III and IV as indicative of difficult laryngoscopy. Univariate and multivariate logistic regression analyses were performed to identify reliable indicators for predicting difficult laryngoscopy. Receiver Operating Characteristic (ROC) curve analysis was utilized to assess the predictive performance of each indicator.

**Results:**

A total of 1120 patients finished the study, with 710 cases found in Grade I laryngoscopic exposure group, 360 cases in Grade II group, and 50 cases in Grade III group. There was no case observed in Grade IV group, thereby resulting in an incidence of difficult laryngoscopy of 4.46%. Univariate logistic regression analysis revealed that several parameters including age, Body Mass Index (BMI), neck circumference, neck mobility, snoring intensity, as well as ultrasound measurements of the pre-epiglottic space and thyromental distance were identified as significant risk factors for difficult laryngoscopy (*P* < 0.05). Among these, BMI, and neck circumference exhibited notable predictive value, with Area Under The Curve (AUC) values of 0.746 (95%CI 0.649–0.842) and 0.732 (95%CI 0.638–0.827), respectively. Neck mobility was also identified as an independent risk factor for predicting difficult laryngoscopy (*P* = 0.009) in multivariate logistic regression analysis, with an AUC of 0.672 (0.562–0.782) in the ROC curve.

**Conclusions:**

Our findings revealed a direct correlation between difficult laryngoscopy and age, BMI, neck circumference, neck mobility, snoring intensity, as well as ultrasound measurements of the pre-epiglottic space and thyromental distance. Furthermore, neck mobility was identified as an independent predictive factor.

**Trial registration:**

The trial was registered prior to patient enrollment at clinicaltrials.gov (register no. ChiCTR2100053826, Date of registration: November 30, 2021).

**Supplementary Information:**

The online version contains supplementary material available at 10.1186/s12871-024-02543-4.

## Introduction

Difficult airway management failure is the primary cause of anesthesia-related fatalities [1–4]. Challenging airway scenarios include difficulties in mask ventilation, supraglottic airway ventilation, supraglottic tool insertion, laryngoscopic exposure, difficult tracheal intubation and recurrent intubation failures [3]. Notably, difficult laryngoscopic exposure has emerged as a key factor leading to unsuccessful management of difficult airways. Difficult laryngoscopy hinges on several factors, including the patient’s airway anatomy, airway management tools, and the anesthesia provider’s expertise with patient related factors acting as uncontrollable variables. Inadequate preoperative airway assessment hinders the clinical management of difficult airways cases, emphasizing the critical need for accurate identification of difficult airways.

Currently, no definitive and reliable indicators are available to predict a difficult airway. The eleven traditional indicators recommended by the American Standards Association (ASA) for airway assessment can effectively predict less than 10% of difficult airways [5]. Furthermore, recognizing the limitations of each method, it becomes imperative to combine the various assessment approaches to enhance the accuracy and sensitivity of predictions [6]. In recent years, ultrasound technology has gained substantial popularity in the field of anesthesia. It is possible to achieve real-time, dynamic airway imaging by placing an ultrasound probe in various positions in the anterior neck. In addition, by measuring relevant anatomical structures, ultrasound can predict the risk of difficult airways, thereby complementing conventional assessment methods.

The objective of this study is to determine the effective predictors of difficult laryngoscopic exposure through a comprehensive approach that integrates medical history analysis, physical examination, and ultrasound examination of the upper airway. The aim was to provide clinicians with novel and reliable predictive methods for assessing difficult laryngoscopy, thereby enhancing patient safety and the efficacy of airway management.

## Methods

### Study subjects

This study was approved by the Ethics Committee of Zhongda Hospital, Southeast University (Approval No: 2021ZDSYLL205-P01) and written informed consent was obtained from all subjects participating in the trial. The trial was registered prior to patient enrollment at clinicaltrials.gov (https://www.chictr.org.cn/showproj.html?proj=129056, Principal investigator: Jing Yuan, Date of registration: November 30, 2021).

The study included patients between the ages of 18 and 75 who were undergoing general anesthesia with endotracheal intubation. They were also classified as ASA grade I to III. However, patients with pre-existing conditions such as head and neck facial trauma, tumors of the maxillofacial region and upper airway, restricted cervical spine mobility, as well as with history of difficult airways were excluded from the study.

### Assessment indicators

#### Medical history and upper airway physical examination indicators

The demographic data including gender, age, height, weight, snoring intensity, and results of upper and lower lip bite tests were recorded. The measurements were collected for the neck circumference, neck mobility, mouth opening capacity, modified Mallampati classification, and hyomental distance.

1. Snoring intensity (None/Mild/Moderate/Severe): Snoring was categorized into three levels based on frequency and volume [7]. No snoring; Mild: Frequency ≤ 2 times/week, volume louder than breathing or similar to speaking; Moderate: Frequency 3–5 times/week, volume louder than speaking; Severe: Frequency 6–7 times/week, audible even through a closed door.

(2) Upper and lower lip bite test (1/2/3): Biting the upper lip with lower incisors extending beyond the upper lip in a calm state without any foreign objects in the mouth was classified as grade 1; lower incisors can touch the upper lip, but should remain below the upper lip line as grade 2; inability of the lower incisors to touch the upper lip is grade 3.

(3) Neck circumference (cm): It was measured from the upper edge of the seventh cervical vertebra at the back to below the thyroid cartilage at the front.

(4) Neck mobility (< 80°/≥80°): It refers to the degree of motion in the cervical spine and the occipito-atlanto joint. The normal range of motion for the neck in the combined extension and flexion ranges is at least 80 degrees or larger.

(5) Thyromental distance ratio (cm/cm): The ratio of distance from the midpoint of the patient’s thyroid cartilage to chin was measured during the neutral and maximum head extension.

(6) Modified Mallampati classification (1/2/3/4): The patient was instructed to open the mouth and extend the tongue as far as possible. Thereafter, classification was performed based on the visibility of pharyngeal structures: Grade I: Soft palate, uvula, and faucial pillars were visible. Grade II: Soft palate, uvula, and partial view of the faucial pillars were obscured by the base of the tongue. Grade III: Only the soft palate was visible. Grade IV: Soft palate was not visible.

### Ultrasound measurement of upper airway parameters

Patients were positioned supine without a pillow upon entering the operating room. An ultrasound of the upper airway was performed using a SonoSite Edge ultrasound machine before induction of anesthesia. A convex array transducer was used to measure the tongue width, tongue thickness, and tongue root distance. A linear array transducer was employed to measure epiglottis depth, epiglottis anterior area, ratio of the thyroid hyoid membrane distance, and mandibular joint mobility.The collection of ultrasound data was performed by a trained physician.

**Fig. 1:**
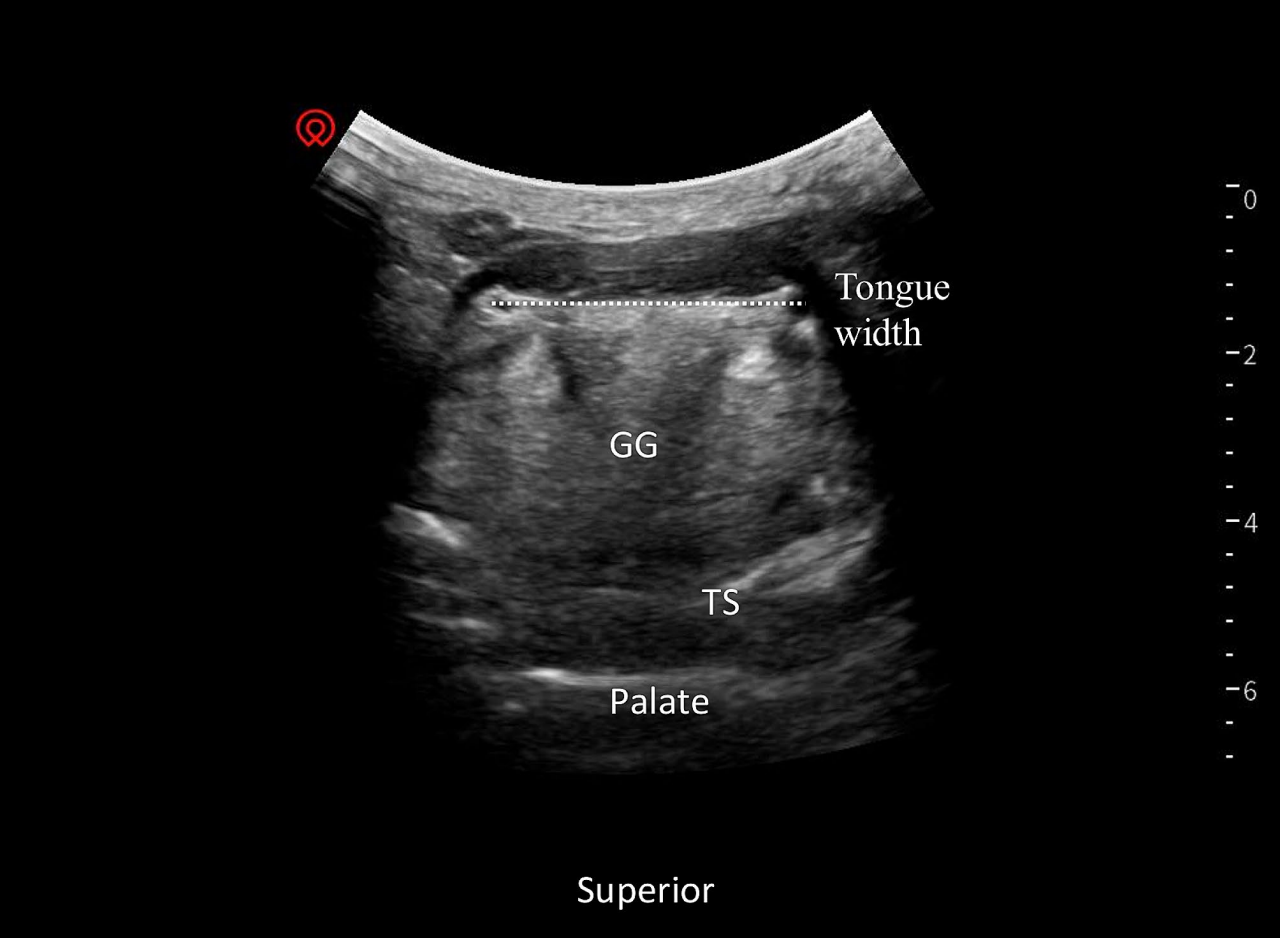
Tongue width. Transverse view of the tongue with low frequency linear transducer in the coronal position below the patient’s mandible, both sides of the lingual arteries were scanned. The maximum distance between the two lingual arteries was measured, as indicated by the dotted line. GG – Genioglossus muscle, TS – Dorsal tongue surface

**Fig. 2:**
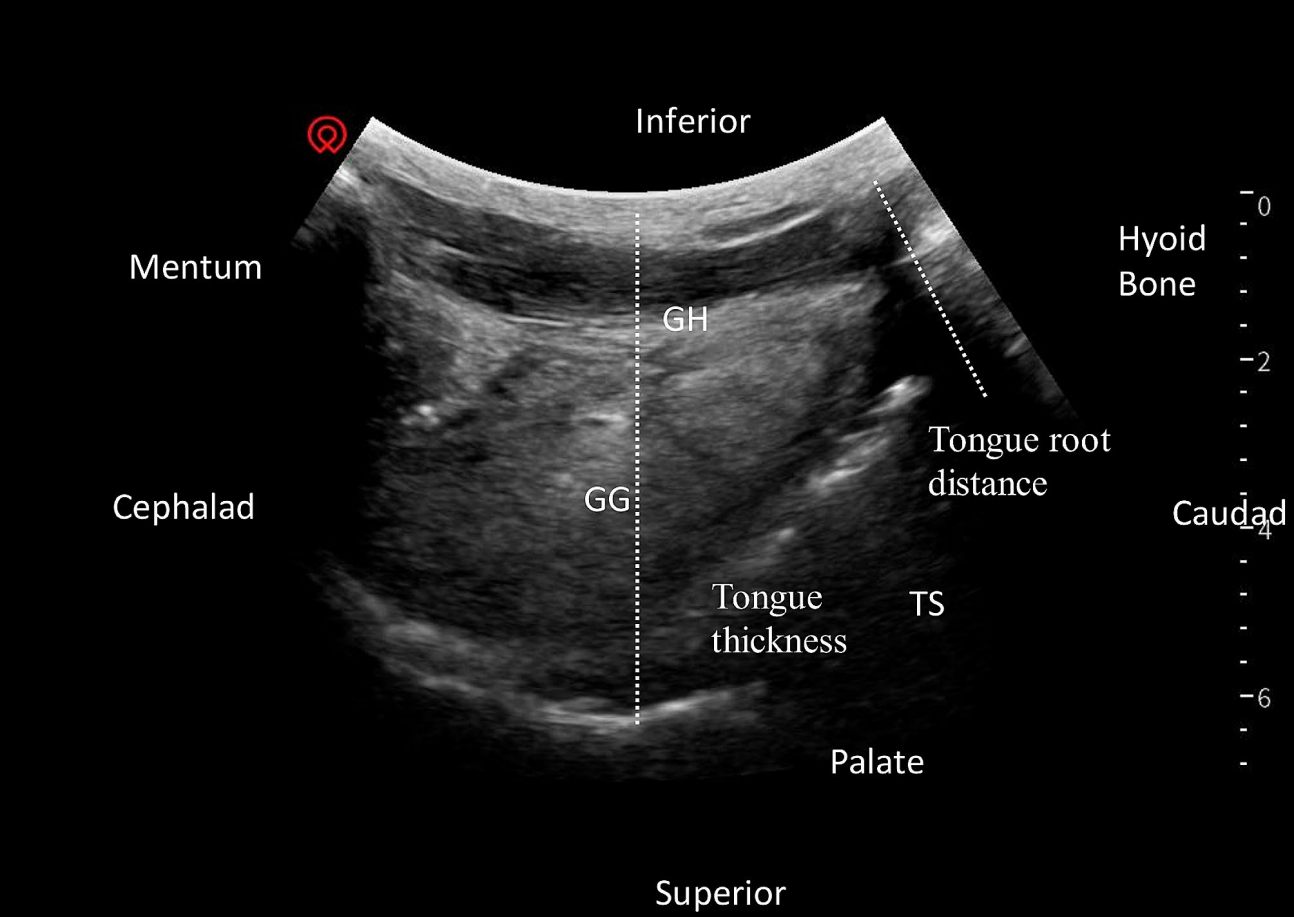
Tongue root distance and Tongue thickness .Sagittal view of the suprahyoid structures using low frequency transducer, placed in the submandibular area behind the mentum. Tongue root distance was measured from the neck’s skin to the tongue root, as indicated by the dotted line. Tongue thickness was measured from the neck’s skin to the farthest end of the tongue, as indicated by the dotted line. GH – Geniohyoid muscle, GG – Genioglossus muscle, TS – Dorsal tongue surface

**Fig. 3:**
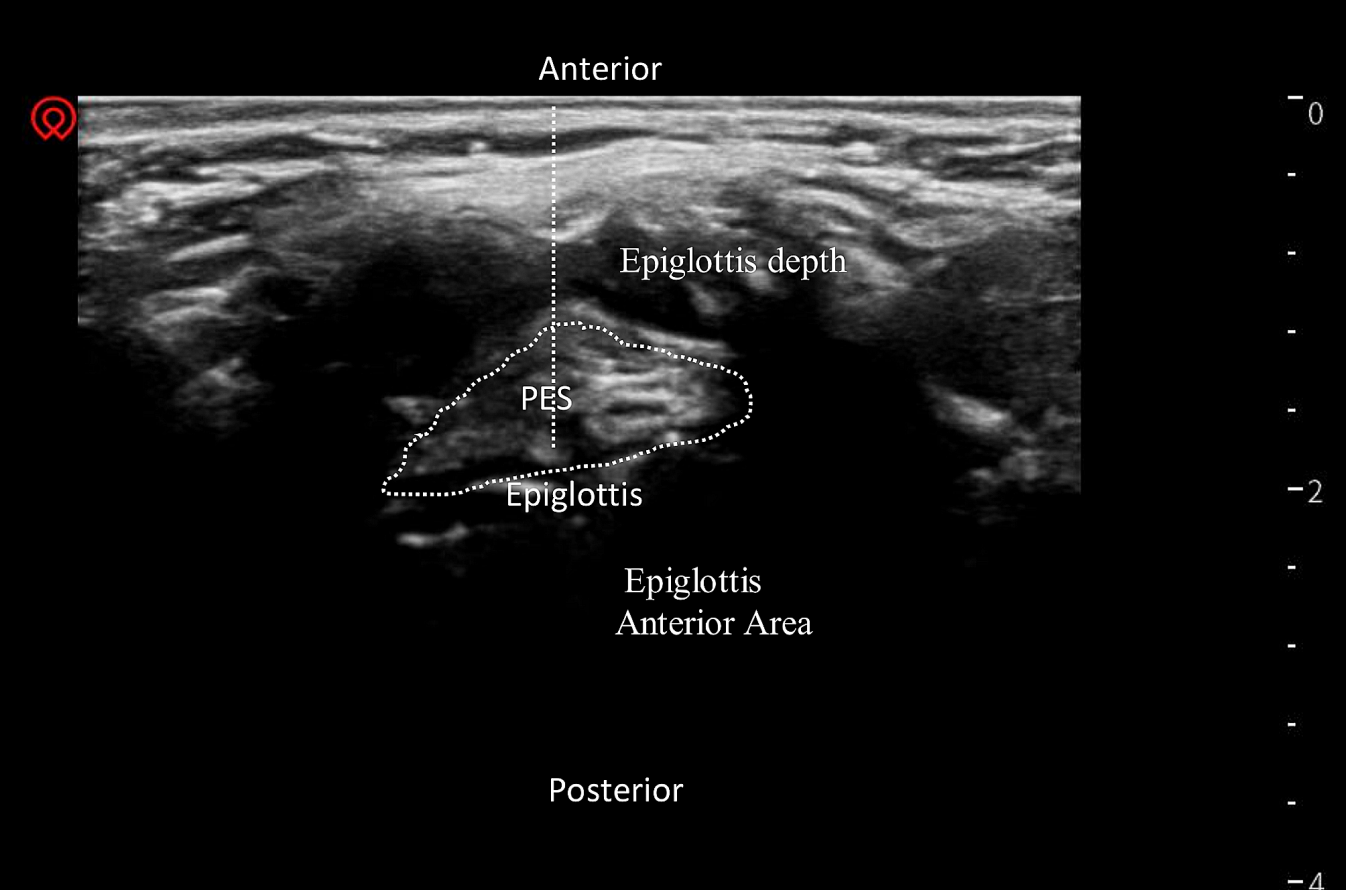
Epiglottis depth and Epiglottis Anterior Area. Transverse view of the epiglottis with high frequency linear transducer over the thyrohyoid area. Epiglottis depth was measured from the midline of the epiglottis to the skin surface, as indicated by the dotted line. Epiglottis Anterior Area was measured, as shown in the graph. PES - Pre-epiglottic space

**Fig. 4:**
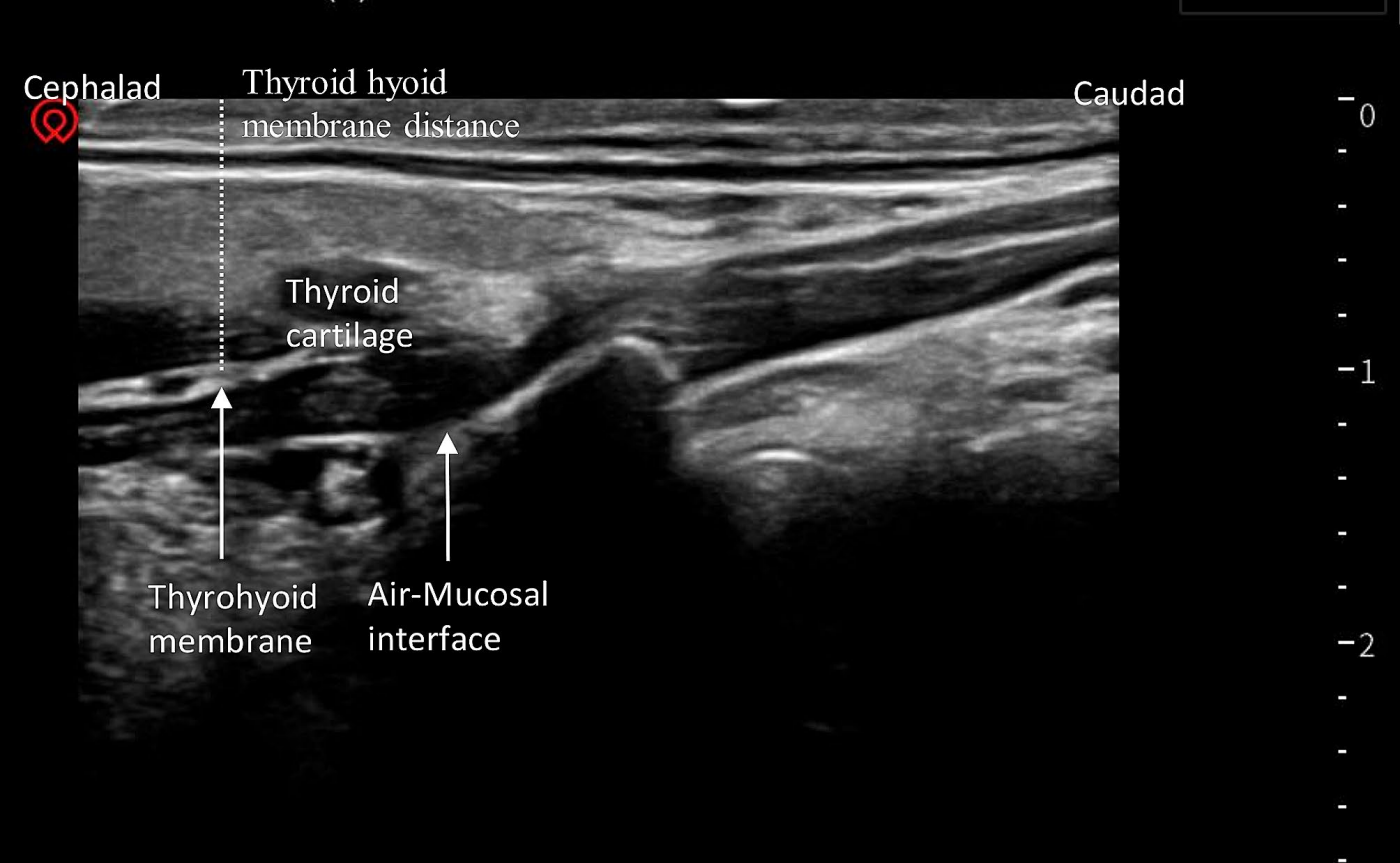
Thyroid hyoid membrane distance. Parasagittal view of the thyrohyoid membrane with high frequency linear transducer. The distance from the bright line to the skin surface was measured, as indicated by the dotted line

**Fig. 5:**
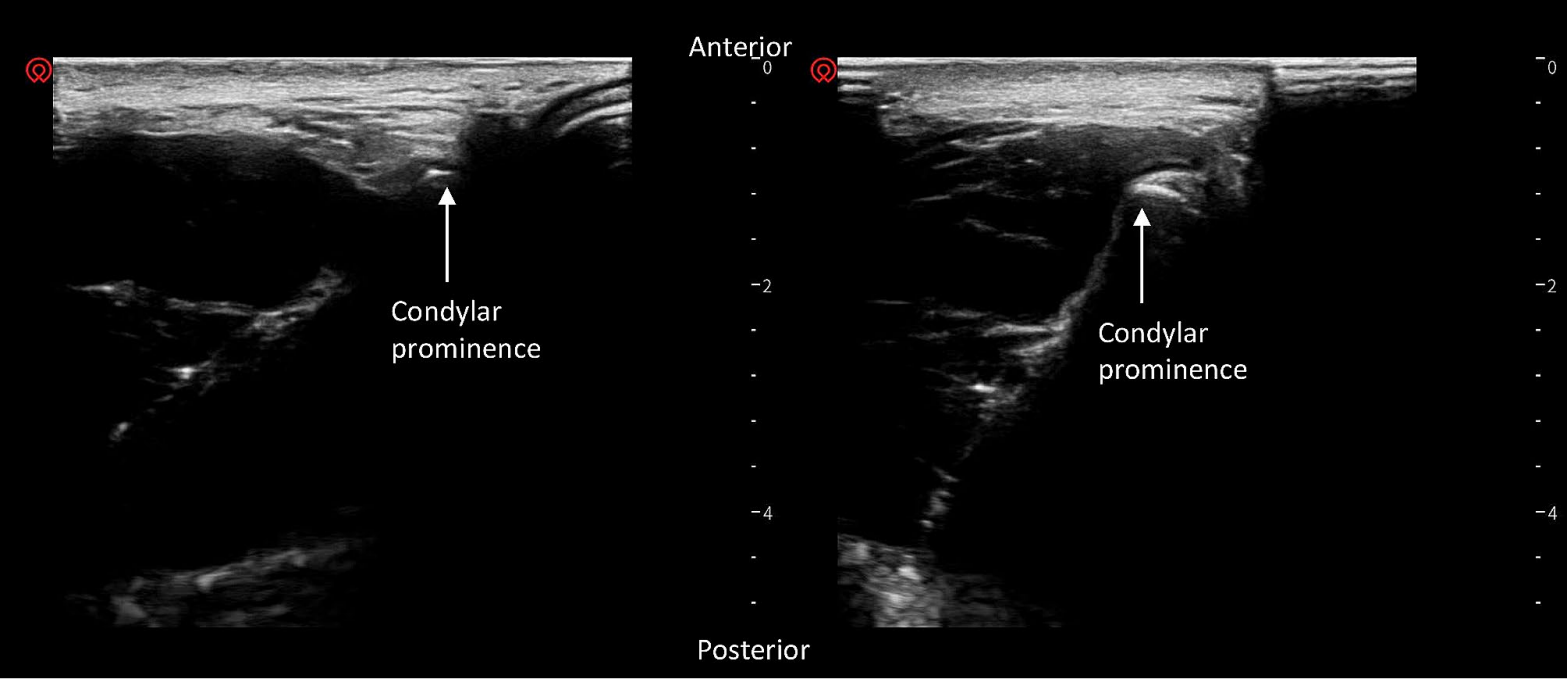
The view of the mandibular joint with high frequency linear transducer. The image of the condylar prominence (high-pointed arc-shaped echo) was captured during mouth opening and closing. The frozen images were compared between the open and closed positions and the distance of condylar prominence sliding was measured

1. Tongue width (cm): The convex array transducer was placed in the coronal position below the patient’s mandible, both sides of the lingual arteries were scanned, and the maximum distance between the two lingual arteries was measured (Fig. 1).

(2) Tongue thickness (cm) : The convex array transducer was placed in the sagittal position below the neck’s mandible for measuring the distance from the neck’s skin to the farthest end of the tongue (Fig. 2).

(3) Tongue root distance (cm) : The convex array transducer was placed in the sagittal position below the neck’s mandible for measuring the distance from the neck’s skin to the tongue root (Fig. 2).

(4) Epiglottis depth and Pre-epiglottic Space Area (cm^2^): The linear array transducer was positioned in the coronal plane on the patient’s neck. Thereafter, scanning from the mandible towards the neck, epiglottis was located and the distance from the midline of the epiglottis to the skin surface and the area of the anterior gap of the epiglottis was measured (Fig. 3).

(5) Thyromental Distance (cm) : The linear array transducer was placed in the mid-sagittal position of the neck for locating the hyoid bone and thyroid cartilage. The bright line between them represented the level of the thyroid hyoid membrane. The distance from the bright line to the skin surface was then measured(Fig. 4).

(6) Mentohyoid Distance Ratio (cm/cm): The transducer was placed in front of the patient’s ear, with one end aligning with the external auditory canal and the other end pointing towards the tip of the nose or the philtrum. The patient was instructed to perform an opening movement while maintaining the transducer stationary relative to the patient’s skin. The image of the condylar prominence (high-pointed arc-shaped echo) was captured during the mouth opening and closing. The frozen images were compared between the open and closed positions and the distance of condylar prominence sliding was measured(Fig. 5).

### Anesthesia induction

After entering the room, all patients received intravenous access and electrocardiography, heart rate, blood pressure, and pulse oxygen saturation monitoring. Every patient received pre-oxygenation for 3 min before anesthesia induction. General anesthesia was induced by i.v. midazolam 2 mg, sufentanil 0.3 to 0.5 µg/kg, propofol 1.5 to 2 mg/kg, and rocuronium bromide 0.6 mg/kg. After the patient’s spontaneous breathing diminished, visual laryngoscope (model: TD-C-III, Taizhou Hanchuang Medical Instruments Technology Co., Ltd.) was used to assess laryngoscopic exposure. During laryngoscopy exposure, the head position is allowed to move and the cricoid cartilage is pressurized to obtain optimal laryngoscopy exposure. The anesthesiologist who performed endotracheal intubation and evaluated laryngoscope exposure was blinded to the airway evaluation results.

### Modified Cormack-Lehane grading of laryngoscopy

An anesthesiologist, more than 3 years of experience, conducted visual laryngoscope and assess laryngoscopic exposure by Modified Cormack-Lehane grading score [8]. The grading system relies on the level of visibility of laryngeal structures, with Grade I indicating full visualization of the vocal cords, Grade II showing visibility of the epiglottis and posterior part of the vocal cords, Grade III indicating only the visibility of the epiglottis, and Grade IV denoting no visibility of the epiglottis. Grades I-II represent uncomplicated laryngoscopic exposure, whereas Grades III-IV indicate difficult laryngoscopic exposure.

### Statistical analysis

The analysis conducted was binary logistic regression. The dependent variable is the result of laryngoscope exposure, which is a binary variable. Covariates are observed upper airway indicators. First, a univariate logistic regression analysis was conducted to identify various risk factors. After that, indicators with significant results were included in the multivariate logistic regression analysis to determine independent risk factors. ROC curves were used to validate the predictive performance.

The sample size was calculated using PASS version 15.0. The study determined the sample size based on a 5% incidence of difficult laryngoscopy exposure, a 20% dropout rate, bilateral alpha set at 0.05, and beta set at 0.2. SPSS version 22.0 was employed for the statistical analysis. A significance level of *P* < 0.05 was considered as statistically significant difference. In multivariate logistic regression analysis, the likelihood ratio test was used to evaluate the model hypothesis. Collinearity analysis was used to test for interaction among the factors.

## Results

**Fig. 6:**
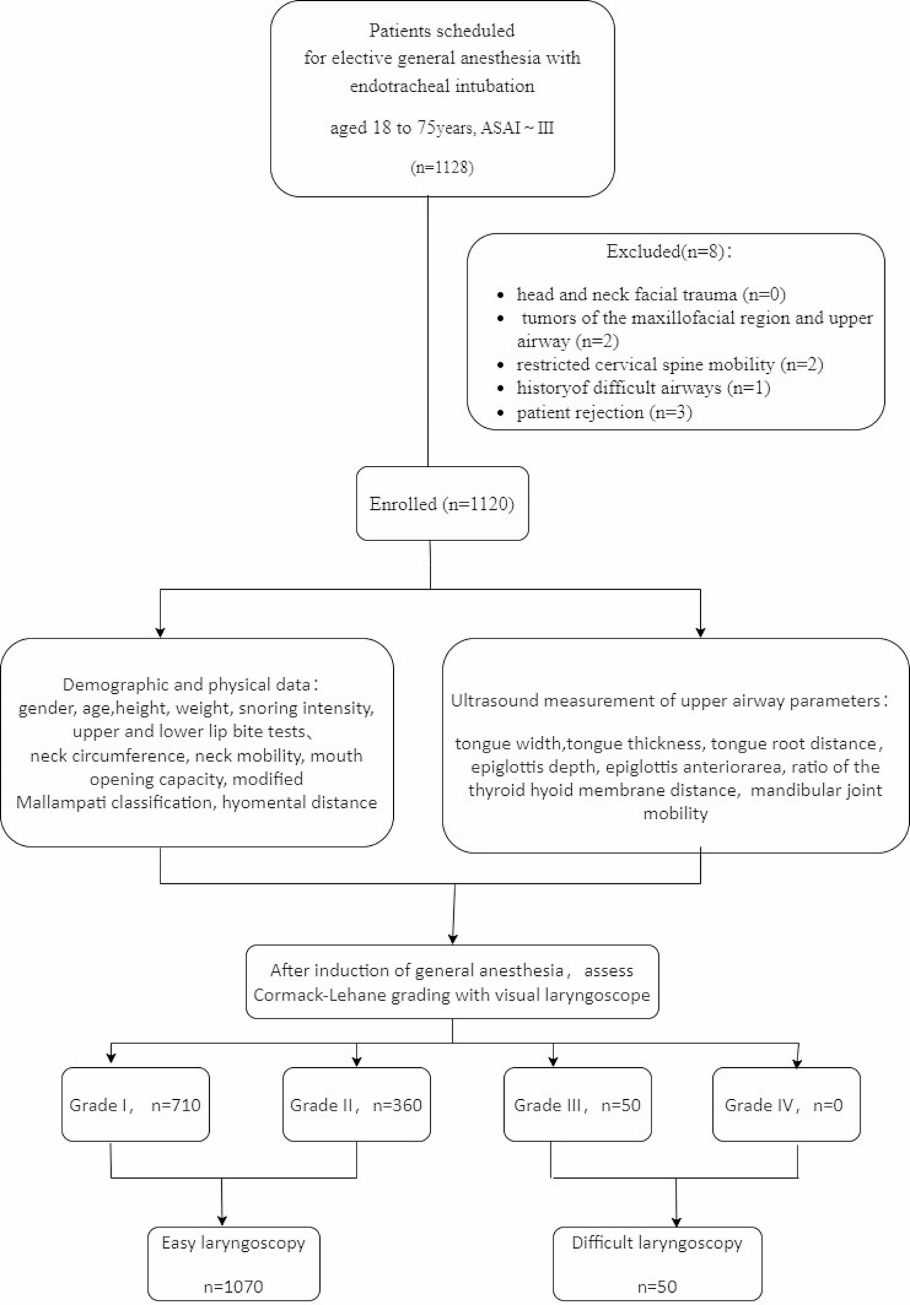
Study flow diagram

We prospectively enrolled 1128 patients scheduled for elective general anesthesia with endotracheal intubation. Five patients were excluded by exclusion criteria. Three patients declined to participate in the study. The study involved 1120 patients ultimately, and their laryngoscopic exposure was classified as grade I in 710 cases, grade II in 360 cases, grade III in 50 cases, and grade IV in 0 cases (Fig. 6). This resulted in a 4.46% incidence of difficult laryngoscopy. Patients with difficult laryngoscopic exposure were more likely to be older and fatter and to have a history of hypertension (Table 1). All patients were subjected to tracheal intubation successfully. However, in the difficult laryngoscopy group, 8 patients required a second intubation attempt, while 2 patients required three attempts to achieve successful intubation.

**Table 1:** Baseline patient and clinical characteristics

	Easy laryngoscopic exposure( *n* = 1070 )	Difficult laryngoscopic exposure( *n* = 50 )	*P*-value
Age (years)	51.24 ± 12.27	58.19 ± 10.20	0.02*
Gender (Male/Female)	493/577	26/24	0.05
BMI (kg/m^2^)	23.44 ± 2.80	26.16 ± 2.63	0.00*
ASA physical status (I/II/III)	0/751/319	0/33/17	0.32
Current cigarette smoking (Yes/No)	375/695	19/31	0.63
Current alcohol drinking (Yes/No)	167/903	9/41	0.78
Hypertension (Yes/No)	321/749	32/18	0.02*
Diabetes mellitus (Yes/No)	326/744	12/38	0.08
Hemoglobin ( g·dL^− 1^)	13.20 ± 3.23	14.32 ± 5.71	0.76
Creatinine (mg·dL^− 1^)	0.73 ± 0.11	0.78 ± 0.16	0.38
Urea nitrogen (mg·dL^− 1^)	11.50 ± 3.23	12.20 ± 3.23	0.69
Alanine aminotransferase (U·L ^− 1^)	30.20 ± 9.23	38.20 ± 5.46	0.28
Aspartate aminotransferase (U·L ^− 1^)	35.20 ± 6.23	37.20 ± 4.14	0.75
SpO 2 in room air (%)	97(94 ~ 99)	97(93 ~ 98)	0.30

**Fig. 7:**
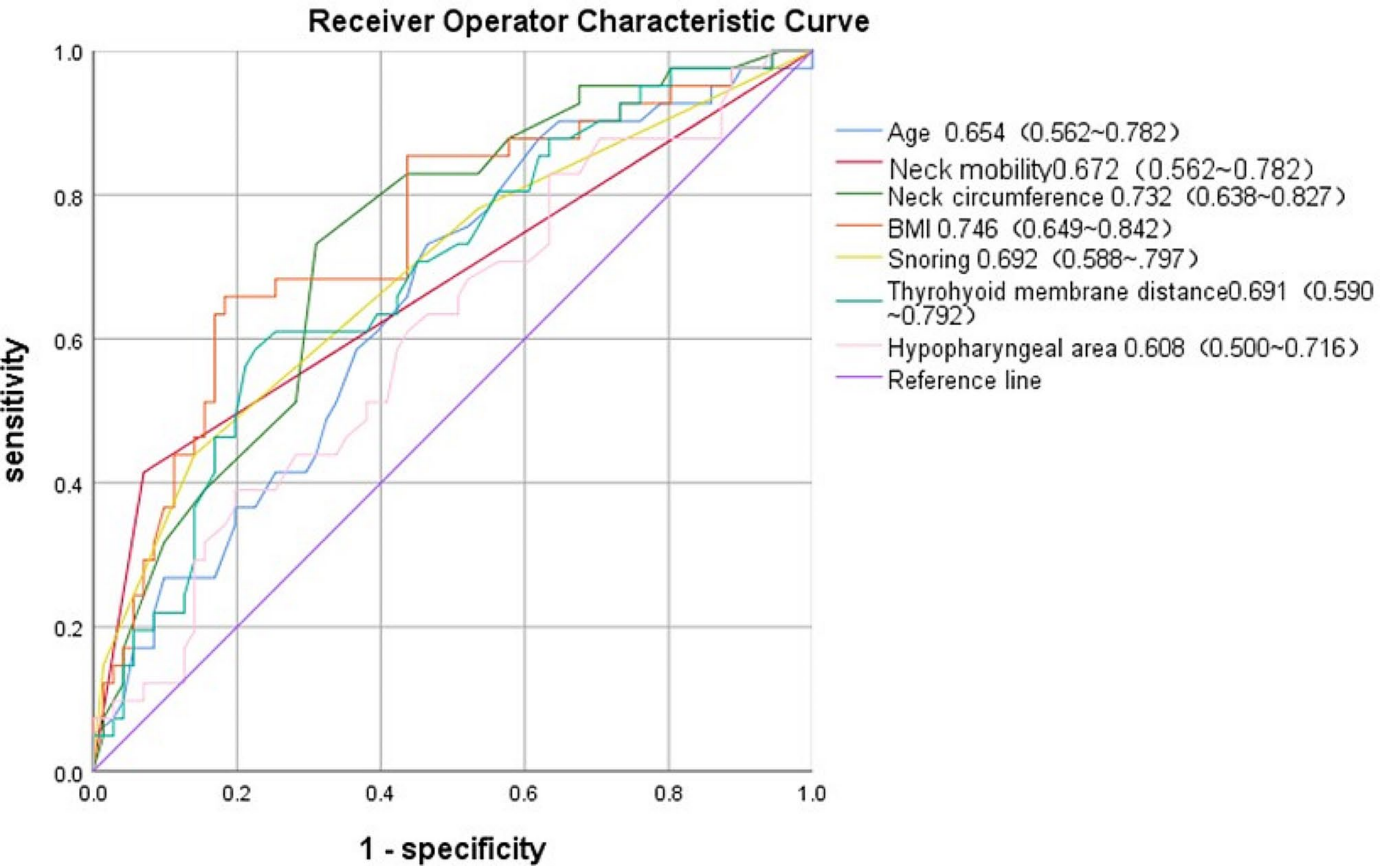
ROC curves for the various risk factors

The results of univariate logistic regression analysis indicated that several parameters, including patient age, BMI, neck circumference, neck mobility, snoring intensity, and ultrasound measurements of the pre-epiglottic space area and thyromental distance, were significant risk factors for difficult laryngoscopic exposure (*P* < 0.05) (Table 2). BMI and neck circumference exhibited good predictive performance, with AUCs of 0.746 (0.649–0.842) and 0.732 (0.638–0.827), respectively (Fig. 7).

**Table 2:** Univariate logistic regression analysis results for difficult laryngoscopic exposure

	Easy laryngoscopic exposure		Difficult laryngoscopic exposure		Odds Ratio (OR)	95% CI of OR		*P*-value	
	Pharyngeal exposure Grade I *n* = 710	Pharyngeal exposure Grade II *n* = 360	Pharyngeal exposure Grade III *n* = 50	Pharyngeal exposure Grade IV *n* = 0					
Age (years)	51.24 ± 12.27		58.19 ± 10.20		1.05	1.01	1.08	0.02*	
Gender (Male/Female)	493/577		26/24		2.17	0.99	4.74	0.05	
BMI (kg/m^2^)	23.44 ± 2.80		26.16 ± 2.63		1.37	1.17	1.61	0.00*	
Neck circumference (cm)	36.80 ± 3.58		40.10 ± 4.34		1.28	1.13	1.46	0.00*	
Neck mobility (< 80°/≥80°)	993/77		29/21		0.11	0.04	0.32	0.00*	
Thyromental Distance Ratio (cm/cm)	1.43 ± 0.15		1.36 ± 0.16		0.04	0.00	0.67	0.06	
Mallampati Grade (1/2/3/4) Mallampati Grade 1 Mallampati Grade 2 Mallampati Grade 3	151/739/180/0		3/35/11/1		0.00 0.00 0.00	0.00 0.00 0.00		0.37 1.00 1.00 1.00	
Upper Lip Bite Test (1/2/3) Upper Lip Bite Test 1 Upper Lip Bite Test 2	708/272/90		24/18/8		0.43 0.83	0.12 0.22	1.48 3.13	0.19 0.18 0.79	
Snoring (None/Mild/Moderate/Severe) Snoring 1 Snoring 2 Snoring 3	498/422/135/15		11/17/14/8		0.05 0.08 0.22	0.01 0.01 0.02	0.43 0.76 2.19	0.01* 0.01 0.03 0.20	
Tongue Width(cm)	3.25 ± 0.37		3.31 ± 0.44		1.38	0.53	3.58	0.51	
Tongue Thickness(cm)	5.96 ± 0.58		6.20 ± 0.46		2.27	0.87	4.82	0.09	
Tongue Base Distance(cm)	3.97 ± 1.21		4.14 ± 0.59		1.12	0.57	2.22	0.74	
Mentohyoid Distance Ratio (cm/cm)	1.43 ± 0.37		1.26 ± 0.16		1.62	0.14	18.34	0.07	
Epiglottis Distance(cm)	2.06 ± 0.40		2.43 ± 0.14		2.03	0.75	5.50	0.11	
Pre-epiglottic Space Area(cm^2^)	0.89 ± 0.11		1.11 ± 0.35		3.47	1.06	11.41	0.04*	
Mandibular Joint Mobility Ratio (cm/cm)	1.41 ± 0.21		1.25 ± 0.14		0.30	0.05	1.78	0.22	
Thyromental Distance(cm)	1.69 ± 0.34		1.94 ± 0.37		7.06	2.12	23.57	0.00*	

The multivariate logistic regression model’s likelihood ratio test showed statistical significance (*P* = 0.00), indicating at least one covariate was predictive. The collinearity test of multivariate logistic regression analysis revealed no significant interaction among covariates (*P* = 0.70) (Table 3). Interestingly, multivariate logistic regression analysis identified neck mobility as an independent predictor of difficult laryngoscopic exposure (*P* = 0.009), with an AUC of 0.672 (0.562–0.782) (Table 4).

**Table 3:** Multivariate logistic regression model fit test and Parallel line test

Model likelihood ratio test					Parallel line test				
**Model**	**log** ^**− 2**^	**F-value**	**V-value**	**P-value**	Model	**log** ^**− 2**^	F-value	V-value	**P-value**
**intercept**	**177.54**				Original	137.40			
**ultimate**	**137.40**	**40.13**	**9**	0.00*	routine	131.05		9	0.70

**Table 4:** Multifactorial logistic regression analysis results for the various risk factors

	AUC	95% Confidence Interval for AUC		OR	95% Confidence Interval for AUC		*P*-value
Neck mobility (< 80°/≥80°)	0.67	0.56	0.78	0.17	0.05	0.65	0.01*
Age (years)	0.65	0.55	0.76	1.02	0.98	1.07	0.34
BMI (kg/m^2^)	0.745	0.65	0.84	1.21	0.98	1.49	0.08
Neck circumference (cm)	0.73	0.64	0.83	1.08	0.88	1.32	0.47
Snoring (None/Mild/Moderate/Severe) Snoring 1 Snoring 2 Snoring 3	0.69	0.59	0.80	0.35 0.30 0.84	0.02 0.02 0.06	5.84 4.27 11.10	0.51
Pre-epiglottic Space Area (cm^2^)	0.61	0.50	0.72	0.81	0.15	4.260	0.80
Thyromental Distance(cm)	0.69	0.59	0.79	3.15	0.72	13.79	0.13

## Discussion

The study results showed that the incidence of laryngoscope exposure difficulties was 4.46%, which is consistent with previous reports.The frequency of difficult airways during the perioperative period is reported to be between 1.5% and 13% [9]. According to ASA statistics from 1970 to 2007, out of 8,984 anesthesia-related cases, difficult intubation was observed in 466 instances, thereby constituting an estimated incidence of 5% for difficult laryngoscopic exposure. All cases were classified as Cormack-Lehane grade III, with no cases classified as grade IV. This may be potentially attributed to the enhanced visibility offered by the visual laryngoscope, featuring a broader angle at the front of the blade for easier epiglottis elevation and glottis exposure.

Neck ultrasound examination can provide clear visualization of upper airway anatomical structures, including the tongue, hard palate, epiglottis, uvula, vocal cords, cricoid membrane, cricoid cartilage, and the anterior wall of tracheal cartilage rings [10]. However, due to the inability of ultrasound to penetrate the gases, various structures located behind the gas in the airway cannot be effectively visualized, including the posterior pharyngeal wall, posterior commissure, and the posterior tracheal wall [11]. Ultrasound measurements have demonstrated greater accuracy in predicting difficult airways compared to conventional methods, exhibiting higher sensitivity and specificity [12]. We have measured various parameters such as tongue width, tongue thickness, tongue root distance, epiglottis depth, pre-epiglottic space area, distance to the hyoid membrane, and the ratio of mandibular joint mobility, which are considered as indicators potentially related to difficult airways. The findings related to ultrasound measurements of soft tissue thickness at distinct positions in the anterior neck for predicting difficult airways are rather inconsistent. For instance, in obese patients (with BMI > 35), Ezril et al. [13] reported that patients with difficult laryngoscopic exposure had greater thickness of horizontal anterior neck soft tissue compared to those with non-difficult exposure, but the results reported by Komatsu et al. [14] were contrasting in nature. In addition, Adhikari et al. [15] found that the distance to the hyoid membrane could effectively predict difficult laryngoscopic exposure. Moreover, Pinto et al. [16] observed a significant correlation between pre-epiglottic space depth and difficult laryngoscopic exposure, with an accuracy of 74.3%, sensitivity of 64.7%, and specificity of 77.1%. When combined with the Mallampati grade, the predictive power improved even further. In our study, pre-epiglottic space area and distance to the hyoid membrane emerged as potential risk factors for difficult laryngoscopic exposure, although their effectiveness in predicting such difficulty is relatively limited.

Obese patients, characterized by a high BMI, often exhibit fat accumulation around the neck, resulting in an increased neck circumference. Additionally, obesity may cause hypertrophy of the tongue, narrowing of the pharyngeal airway and the occurrence of snoring symptoms of different intensities. Consequently, obese patients have a higher likelihood of experiencing difficult airways. Our study investigated various indicators associated with obesity such as BMI, neck circumference and the degree of snoring as potential risk factors for difficult laryngoscopic exposure. Among these, BMI, and neck circumference demonstrated relatively good predictive efficacy. however, there was no correlation found between difficult laryngoscopic exposure and ultrasound measurements of tongue width, thickness, and root distance. Thus, it is possible that these indicators could be more relevant to difficulties in mask ventilation.

Both the upper lip bite test and ultrasound measurements of mandibular joint mobility reflect the ability of the mandible’s movement capability. It has been reported that restricted mandibular mobility increases the likelihood of difficult laryngoscopic exposure [17]. While previous studies found that the upper lip bite test could accurately predict the risk of difficult laryngoscopic exposure [18], our study did not observe a potential correlation between difficult laryngoscopic exposure and either of these indicators. This may be attributed to the use of a visual laryngoscope with a larger angle at the front end of the blade in our study, effectively overcoming the challenges posed by restricted mandibular mobility.

Our findings suggest that age is also a relevant factor in difficult laryngoscopic exposure, likely due to the increased probability of obesity with age. Additionally, aging is associated with bone proliferation, causing ligament calcification and a reduction in cervical joint mobility. This can lead to the restricted neck movement, thus resulting in an increased probability of difficult laryngoscopic exposure. Age is therefore a manifestation of the combined effects of several factors.

The ratio of the thyromental distance measured at the neutral head position and the maximum head extension is regarded a traditional indicator for predicting difficult airways using surface measurements. Both the ultrasound-measured tongue-mentum distance ratio and the thyromental distance ratio effectively reflect neck mobility. For instance, Wojtczak in his study observed six patients with a history of difficult laryngoscopic exposure and observed a significant difference in their tongue-mentum distance ratio in comparison to patients without exposure difficulties (*P* < 0.002) [19]. Our findings indicate that reduced neck mobility can serve as a potential risk factor for difficult laryngoscopic exposure, and is an independent predictive factor. However, we did not observe a correlation between the thyromental distance ratio, tongue-mentum distance ratio, and difficult laryngoscopic exposure. This result was consistent with clinical observations, demonstrating that restricted neck movement in conditions like ankylosing spondylitis and fusion of cervical facet joints can often lead to difficult laryngoscopic exposure.

It should be noted that this study only included participants from the normal general population. Special groups, such as patients with obesity or limited neck mobility, may be more susceptible to difficulties during laryngoscopy. It would be useful to perform stratified analysis on these groups, expanding the sample size, in order to achieve more clinical significance in the future.

Recognizing a difficult airway is crucial for effectively managing the difficult airways and predicting difficult airways could be an enduring as well as challenging task for anesthesiologists. The ongoing evolution of ultrasound technology offers substantial promise in predicting difficult airways, and its integration with conventional approaches can markedly improve the accuracy of predictions. Further research involving larger sample sizes and multicenter studies is needed to validate the prediction of difficult airways. This would involve the examination of diverse indicators in order to provide more comprehensive guidance for clinical practice.

## Conclusions

Our findings revealed a direct correlation between difficult laryngoscopy and age, BMI, neck circumference, neck mobility, snoring intensity, as well as ultrasound measurements of the pre-epiglottic space and thyromental distance. Furthermore, neck mobility was identified as an independent predictive factor.

### Electronic supplementary material

Below is the link to the electronic supplementary material.

Supplementary Material 1

## Data Availability

The datasets used and/or analysed during the current study are available from the corresponding author on reasonable request.

## Abbreviations

ASA: American Standards Association
BMI: Body Mass Index
AUC: Area Under The Curve
ROC: Receiver Operating Characteristic
OR: Odds Ratio
