# Supplementary material for: Determinants of difficult laryngoscopy based on upper airway indicators: a prospective observational study
Source: BMC Anesthesiol. 2024 Apr 24;24:157. doi: 10.1186/s12871-024-02543-4 (PMC11040868; doi:10.1186/s12871-024-02543-4)
Supplement: Supplementary file 1 — Supplementary Material 1 [file 12871_2024_2543_MOESM1_ESM.pdf]

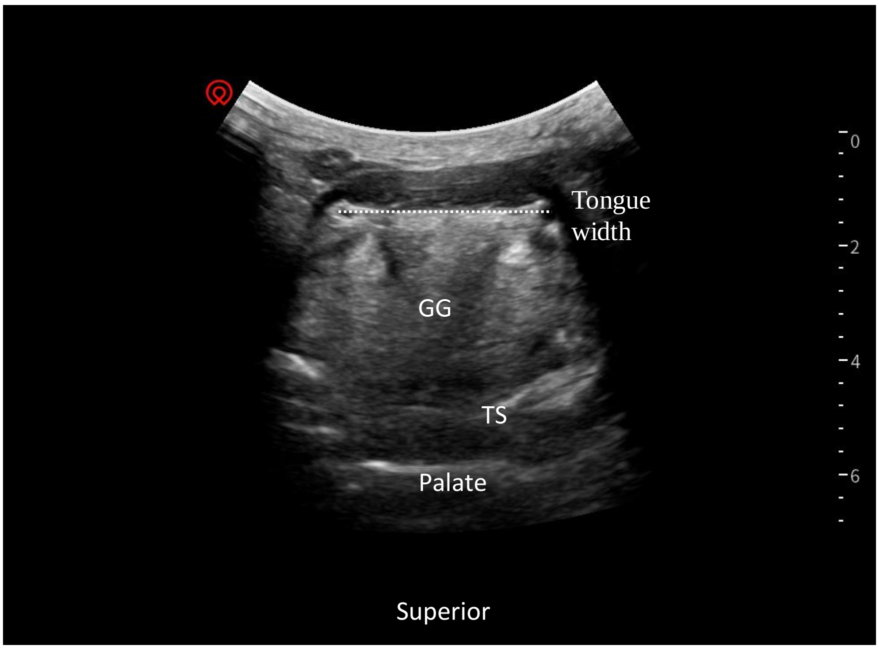

Figure 1. Tongue width. Transverse view of the tongue with low frequency linear transducer in the coronal position below the patient's mandible, both sides of the lingual arteries were scanned. The maximum distance between the two lingual arteries was measured, as indicated by the dotted line. GG – Genioglossus muscle, TS – Dorsal tongue surface.

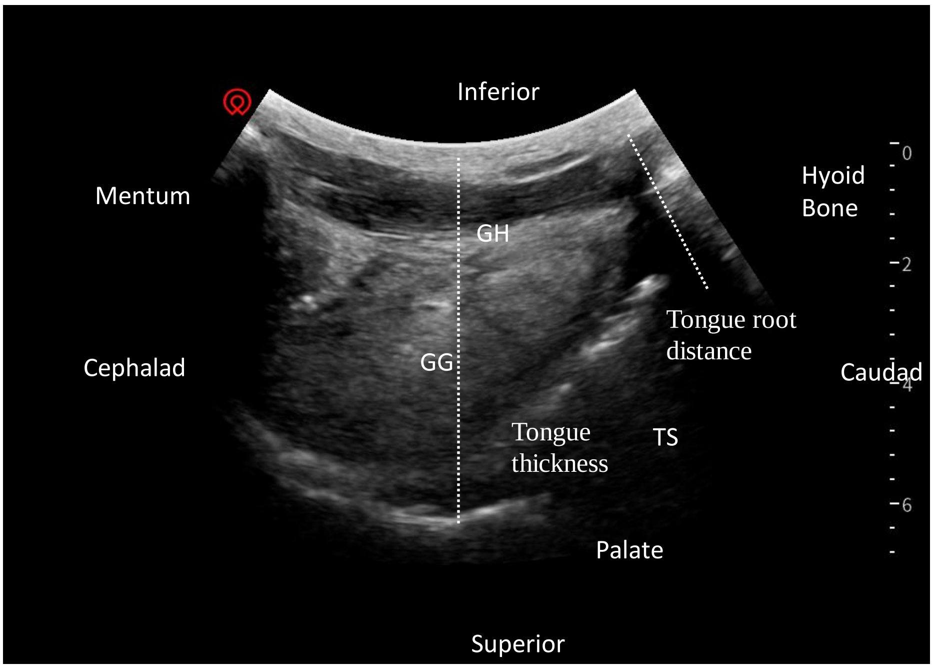

Figure 2. Tongue root distance and Tongue thickness .Sagittal view of the suprahyoid structures using low frequency transducer, placed in the submandibular area behind the mentum. Tongue root distance was measured from the neck's skin to the tongue root, as indicated by the dotted line. Tongue thickness was measured from the neck's skin to the farthest end of the tongue, as indicated by the dotted line. GH – Geniohyoid muscle, GG – Genioglossus muscle, TS – Dorsal tongue surface.

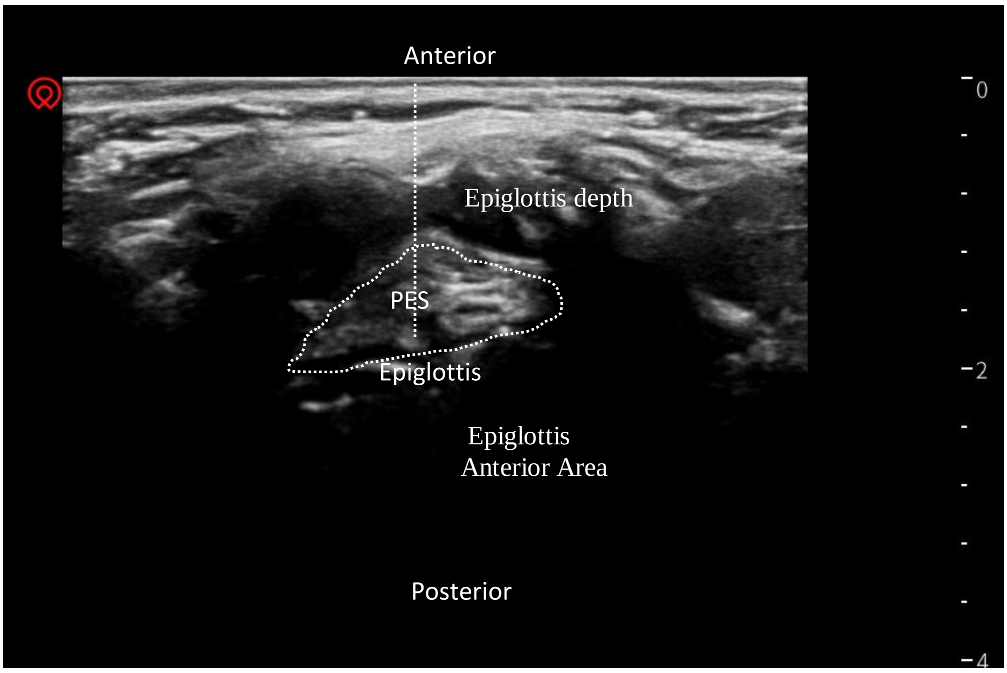

Figure 3. Epiglottis depth and Epiglottis Anterior Area. Transverse view of the epiglottis with high frequency linear transducer over the thyrohyoid area. Epiglottis depth was measured from the midline of the epiglottis to the skin surface, as indicated by the dotted line . Epiglottis Anterior Area was measured, as shown in the graph. PES - Pre-epiglottic space.

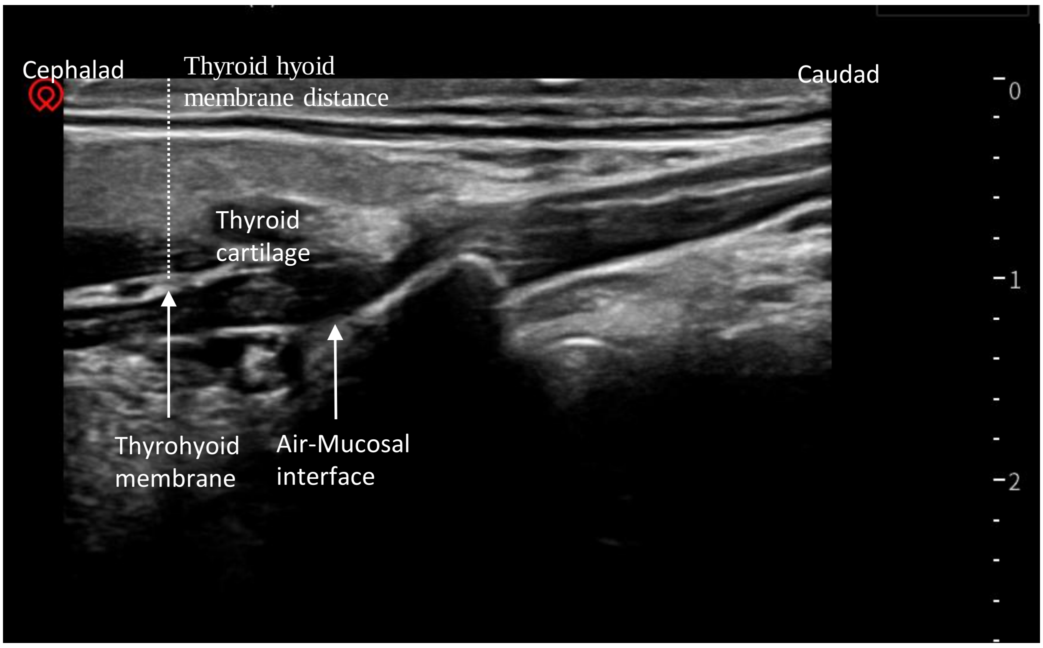

Figure 4. Thyroid hyoid membrane distance. Parasagittal view of the thyrohyoid membrane with high frequency linear transducer. The distance from the bright line to the skin surface was measured, as indicated by the dotted line.

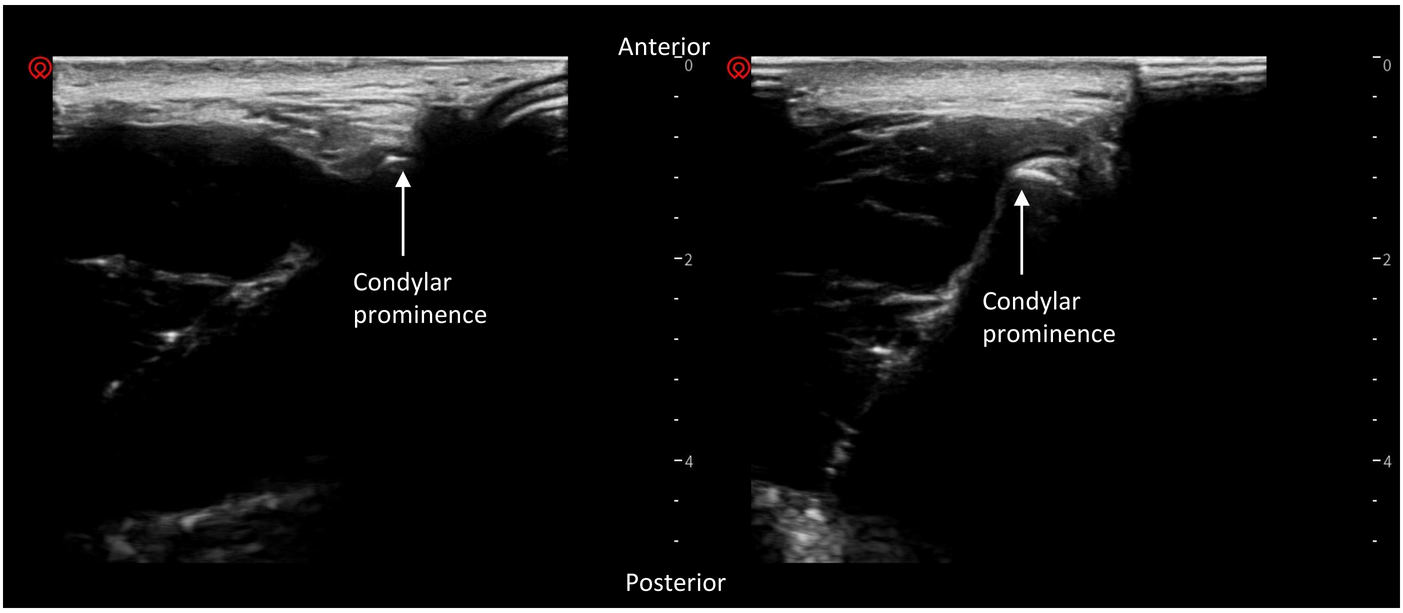

Figure 5. The view of the mandibular joint with high frequency linear transducer. The image of the condylar prominence (high-pointed arc-shaped echo) was captured during mouth opening and closing. The frozen images were compared between the open and closed positions and the distance of condylar prominence sliding was measured.
